# Long-Term Outcome in Implant Breast Reconstruction and Radiotherapy: The Role of Fat Grafting

**DOI:** 10.3390/jcm14217569

**Published:** 2025-10-25

**Authors:** Alessia De Col, Francesco Buttarelli, Melissa Akuma, Ferruccio Paganini, Anna Scevola

**Affiliations:** 1 Ospedale Sant’Anna di Como, ASST-Lariana, 22100 Como, Italy; alessia.decol@asst-lariana.it (A.D.C.);; 2 Division of Plastic and Reconstructive Surgery, University of Padova, 35121 Padova, Italy; 3 Division of Plastic and Reconstructive Surgery, Department of Biotechnology and Life Sciences, University of Insubria, 21100 Varese, Italy; fpaganini@studenti.uninsubria.it; 4 Division of Plastic and Reconstructive Surgery, University of Federico II, 80126 Napoli, Italy; melissa.akuma@asst-lariana.it

**Keywords:** breast reconstruction, fat grafting, radiotherapy, implant

## Abstract

**Background:** Capsular contracture remains one of the most challenging complications of implant-based breast reconstruction, particularly in patients undergoing postmastectomy radiotherapy (PMRT). Autologous fat grafting has been proposed as a regenerative strategy to mitigate radiation-induced damage, but long-term data are limited. **Methods:** We retrospectively reviewed women who underwent two-stage implant-based breast reconstruction followed by PMRT (50 Gy in 25 fractions) between 2010 and 2021 at Ospedale Sant’Anna, Como. Eligible patients subsequently received at least one session of autologous fat grafting after radiotherapy. Primary outcome was the incidence and severity of capsular contracture; secondary outcomes included the need for salvage autologous reconstruction, oncologic safety, and patient-reported satisfaction. **Results:** Thirty-two patients met inclusion criteria. The mean age was 56.1 years, and mean BMI was 23.8 kg/m^2^. All underwent submuscular two-stage reconstruction with anatomically shaped implants (mean volume 336 cc). Patients received an average of 1.7 fat grafting sessions (mean cumulative volume 180 cc). At a mean follow-up of 7.7 years, capsular contracture occurred in 6 patients (18.8%): 4 with Baker grade III and 2 with Baker grade II. No cases of severe (grade IV) contracture were observed. Importantly, no patient required salvage autologous reconstruction, and no local recurrences were recorded. Minor donor-site complications (transient edema or ecchymosis) occurred in 18.7% of patients. Subjective satisfaction was uniformly high, with reported improvements in breast softness and contour. **Conclusions:** Fat grafting appears to be a safe and effective adjunct in maintaining implant-based breast reconstruction after radiotherapy. In this long-term series, lipofilling was associated with a lower incidence of capsular contracture compared with historical rates, absence of severe contracture, and no oncologic events. For selected patients who are not candidates for autologous reconstruction, fat grafting may represent a valuable strategy to preserve implant viability, improve tissue quality, and reduce the need for salvage procedures.

## 1. Introduction

Capsular contracture remains one of the most frequent and challenging complications in implant-based breast reconstruction, particularly in patients who undergo postmastectomy radiotherapy (PMRT). From a biological perspective, any breast implant is recognized as a foreign body, leading to the formation of a fibrous capsule around the prosthesis. While in most patients this reaction is mild and clinically irrelevant, in a subset, the fibrotic response becomes exaggerated, resulting in progressive tightening of the capsule, pain, deformity, and the potential need for surgical revision or implant removal. Reported rates of capsular contracture in the general implant population range from 2.8% to 20.4% [1,2,3,4].

Radiotherapy is a well-established risk factor for capsular contracture, as ionizing radiation induces fibroblast proliferation, chronic inflammation, and disorganized collagen deposition [5,6]. According to a meta-analysis by Ricci et al. [5], capsular contracture develops in 40–50% of irradiated implant reconstructions, with some series reporting even higher rates. Although autologous reconstruction remains the gold standard for patients requiring PMRT—since vascularized tissue transfer effectively replaces irradiated tissues—many women are not candidates for flap-based procedures due to comorbidities, limited donor sites, or personal preference. Consequently, implant-based reconstruction followed by radiotherapy continues to represent a significant clinical scenario, albeit with high complication risks [6,7,8].

In parallel, attention has also been drawn to other risk factors influencing reconstructive outcomes. Beyond radiotherapy, several anatomical and technical variables can affect reconstructive outcomes, including flap thickness and the use of new prosthetic materials and devices. Recent studies have explored these aspects, highlighting ongoing efforts to optimize implant stability and soft-tissue healing despite irradiation [9,10,11,12].

In recent years, autologous fat grafting (lipofilling) has been increasingly investigated as a regenerative strategy to improve the quality of irradiated tissues. Adipose-derived stromal cells (ADSCs) exert angiogenic, antifibrotic, and immunomodulatory effects, which may counteract radiation-induced damage and improve the periprosthetic environment [13]. Several studies have suggested that lipofilling enhances tissue pliability and reduces the incidence of capsular contracture, although the available evidence remains heterogeneous and conflicting. For instance, Martin et al. [14] found no significant protective effect, while other groups have reported encouraging results.

Despite the growing interest, long-term data on the impact of fat grafting in irradiated implant-based reconstructions remain limited. Most published series include short to mid-term follow-up, and oncologic safety concerns have also been raised regarding the potential stimulatory effect of ADSCs in breast cancer survivors.

The present study aims to evaluate the long-term outcomes of fat grafting in women with implant-based breast reconstruction after radiotherapy, with a focus on the incidence of capsular contracture, the need for salvage autologous reconstruction, and oncologic safety. We hypothesized that fat grafting could mitigate the adverse effects of radiotherapy and allow maintenance of implants in a selected group of patients.

## 2. Materials and Methods

### 2.1. Study Design and Patient Selection

We conducted a retrospective review of medical records of women who underwent mastectomy followed by two-stage implant-based breast reconstruction and postmastectomy radiotherapy (PMRT) between January 2010 and December 2021 at Ospedale Sant’Anna di Como (ASST-Lariana). Most mastectomies were performed for invasive carcinoma or extensive ductal carcinoma in situ. All cases involved either skin-sparing mastectomy (SSM) or nipple-sparing mastectomy (NSM), according to tumor location and oncologic safety criteria discussed within the multidisciplinary tumor board. No simple mastectomies were included. Eligible patients subsequently underwent at least one session of autologous fat grafting after the completion of radiotherapy, in line with institutional protocols and previous reports describing the use of lipofilling in irradiated breast reconstructions.

Inclusion criteria were:

- Two-stage implant-based reconstruction with placement of a tissue expander followed by exchange to a definitive implant, a widely adopted technique in clinical practice.
- PMRT delivered on the permanent implant, according to the institutional protocol (50 Gy in 25 fractions), consistent with international oncologic guidelines [15].
- Subsequent autologous fat grafting performed after radiotherapy.
- Minimum follow-up of 3 years, ensuring long-term evaluation of reconstructive outcomes [16].
- Availability of complete clinical data.

Exclusion criteria were:

- Hybrid reconstructions involving autologous tissue components (latissimus dorsi or free flaps), which are known to alter the biological environment and complicate the interpretation of implant-related outcomes.
- Immediate reconstructive failure.
- Early complications attributable to radiotherapy, such as severe flap necrosis or infection, which may independently require salvage autologous procedures [17].
- Incomplete follow-up.
- Different radiotherapy regimen.
- Neoadjuvant or adjuvant chemotherapy during the study period, given their potential confounding effects on local tissue response.

According to institutional policy, radiotherapy is systematically delivered after the exchange to the permanent implant, rather than to the tissue expander, to minimize complications related to expansion during irradiation and to ensure a stable breast volume for accurate dosimetry. Direct-to-implant reconstruction was not adopted in this cohort due to institutional preference for two-stage procedures, particularly in patients scheduled for PMRT, as this approach allows safer soft-tissue adaptation and easier management of post-radiation changes. The interval between definitive implant placement and the start of radiotherapy averaged 6 to 8 weeks (range, 5–10 weeks), allowing complete wound healing and soft-tissue stabilization before PMRT.

This rigorous selection was adopted to ensure a homogeneous study population and minimize variability. Similar methodological approaches have been recommended in systematic reviews evaluating implant-based reconstruction in complex oncologic settings. Furthermore, consideration of risk factors such as mastectomy flap thickness and the use of novel biomaterials has been shown to impact reconstructive outcomes, supporting the importance of strict inclusion and exclusion criteria.

### 2.2. Surgical Technique

All patients underwent a standardized two-stage procedure with submuscular pocket dissection, placement of an anatomically shaped silicone tissue expander (Mentor^®^ (Santa Ana, CA, USA) or Allergan^®^ (Irvine, CA, USA)), and subsequent exchange for a definitive silicone implant of comparable characteristics. All patients underwent a standardized two-stage procedure with complete submuscular pocket dissection, without the use of acellular dermal matrices (ADMs) or meshes. This uniform technique was adopted throughout the study period to ensure comparable implant coverage and minimize variability in capsule formation. The two-stage submuscular approach is widely regarded as a reliable method for implant-based reconstruction, providing soft tissue coverage and facilitating adaptation to postoperative radiotherapy.

Fat grafting was performed using a uniform technique, consistent with protocols previously described in the literature [18]. Harvesting was carried out from the abdominal wall, flanks, or trochanteric areas after infiltration with a tumescent solution (saline, lidocaine, and epinephrine). Lipoaspirates were collected in 10 mL Luer-Lock syringes and centrifuged at 3000 rpm for 3 min to separate the adipose fraction, according to the Coleman technique. Purified fat was then injected in small aliquots with blunt cannulas in a multilayered and radial fashion into the subcutaneous plane of the reconstructed breast, targeting areas of radiation-induced stiffness, contour irregularities, or volume deficiency. The indication for fat grafting was both functional and aesthetic: improving the pliability and vascularity of irradiated tissues to prevent or mitigate capsular contracture, and correcting contour irregularities or asymmetry for cosmetic optimization. This stepwise, micro-aliquot injection method reduces the risk of fat necrosis and promotes graft integration through maximal surface contact with recipient tissues.

The mean number of fat grafting sessions, volume per session, and cumulative injected volume were recorded for each patient. These technical details are clinically relevant, as repeated sessions and cumulative volume have been associated with more favorable outcomes in irradiated breasts. Moreover, fat grafting has been increasingly integrated into prepectoral reconstruction strategies, including those using dermal slings or ADMs, where it serves both regenerative and aesthetic purposes.

### 2.3. Postoperative Follow-Up

Follow-up visits were scheduled at 7 and 15 days postoperatively, then at 1, 3, 6, and 12 months, and annually thereafter. This schedule is consistent with previously published protocols for long-term surveillance of breast reconstruction patients. Each consultation included a standardized clinical assessment focusing on:

- Implant integrity.
- Breast symmetry.
- Presence and severity of capsular contracture, assessed using Baker’s classification [1], which remains the most widely adopted clinical tool despite its subjective limitations.
- Patient-reported satisfaction, obtained through structured clinical interviews. Although validated questionnaires such as the BREAST-Q have been increasingly employed in contemporary research [19,20], at the time of data collection, such tools were not routinely implemented in our institutional practice.

Oncologic surveillance was conducted according to the institutional breast cancer follow-up protocol, including periodic clinical examination and radiological imaging (mammography, ultrasound, and/or MRI when indicated). This multimodal approach is in line with international guidelines [15,21] and ensures both early detection of recurrence and accurate assessment of reconstructed breast tissues. The role of imaging is particularly relevant in patients who have undergone fat grafting, as radiological evaluation can differentiate between benign post-grafting changes and suspicious lesions [22,23,24].

### 2.4. Outcomes and Statistical Analysis

The primary outcome of the study was the incidence and severity of capsular contracture following fat grafting in irradiated implant-based reconstructions. Contracture was assessed clinically using Baker’s classification, which, despite its intrinsic subjectivity, remains the most widely adopted and reproducible tool for long-term evaluation of implant outcomes.

Secondary outcomes included:

- The need for salvage autologous breast reconstruction, an important endpoint considering that autologous conversion is often required in patients with severe post-radiation complications [25,26,27].
- Oncologic safety, defined as the absence of clinically detectable local recurrence during follow-up, in accordance with international breast cancer surveillance standards [23].

Additional observations included patient-reported satisfaction, as subjectively assessed during follow-up consultations. 

Statistical analysis was primarily descriptive, reflecting the relatively small sample size and the exploratory nature of this investigation. All analyses were performed using IBM SPSS Statistics for Windows, version 27.0 (IBM Corp., Armonk, NY, USA). Continuous variables are presented as mean ± standard deviation (SD) and range; categorical variables are expressed as frequencies and percentages. No comparative statistical testing was performed due to the limited cohort size and the risk of type II error. Similar methodological approaches have been reported in previous retrospective series with small patient cohorts, where the focus was hypothesis-generating rather than inferential.

## 3. Results

### 3.1. Patient Characteristics

A total of 32 patients met the inclusion criteria and were included in the analysis. The mean age at surgery was 56.1 ± 10.8 years (range, 37–80), and the mean body mass index (BMI) was 23.8 ± 2.3 kg/m^2^, reflecting a predominantly normal-weight population. The majority of patients were non-smokers, and none had poorly controlled systemic comorbidities at the time of reconstruction, ensuring a relatively homogeneous cohort.

All patients underwent two-stage implant-based breast reconstruction with anatomically shaped silicone-filled implants. The mean implant volume was 335.9 ± 38.5 cc (range, 250–400 cc), with implant sizes chosen to achieve natural symmetry relative to the contralateral breast.

The mean follow-up duration was 7.7 years (range, 3.6–14.7), providing robust long-term data on reconstructive and oncologic outcomes. Demographic and clinical data are summarized in Table 1.

### 3.2. Surgical and Fat Grafting Data

All patients received PMRT with a total dose of 50 Gy delivered in 25 fractions over 5 weeks, in accordance with institutional standards.

Patients underwent on average 1.7 ± 0.6 sessions of fat grafting (range, 1–3), reflecting the need for repeated procedures in a subset of cases to achieve optimal correction of contour irregularities and tissue compliance. The mean volume of fat injected per session was 105 ± 28 cc, with a mean cumulative volume of 180 ± 45 cc per patient, indicating a moderate but consistent amount of grafted tissue across the cohort.

Donor sites most frequently included the abdominal wall (81%), followed by the trochanteric region (56%) and the flanks (34%). In several patients, multiple donor sites were combined to obtain the required volume, highlighting the versatility of harvesting areas.

No major perioperative complications were observed during fat harvesting or injection. Minor complications consisted of transient edema and ecchymosis at the donor sites in 6 patients (18.7%), all of which resolved spontaneously without the need for medical or surgical intervention. Importantly, no cases of fat necrosis, oil cyst formation, or infection were recorded, supporting the safety of the adopted technique.

### 3.3. Capsular Contracture

Capsular contracture occurred in 6 of 32 patients (18.8%) during the follow-up period. Among these, 4 patients (12.5%) developed Baker grade III contracture, associated with breast firmness and mild distortion, while 2 patients (6.3%) developed Baker grade II contracture, characterized by a moderate increase in capsule consistency without significant deformity. Importantly, no cases of Baker grade IV contracture were recorded in this series.

The mean time to diagnosis of contracture was 4.2 years after radiotherapy (range, 2.8–6.7), indicating that this complication typically developed several years after treatment rather than in the immediate postoperative period.

The overall incidence of contracture in our cohort (18.8%) was markedly lower compared with published rates of 40–50% in irradiated implant-based reconstructions. Moreover, the absence of severe grade IV contracture further supports the long-term tolerability of implant maintenance when combined with fat grafting (Table 2). These results are summarized in Table 2, which contextualizes our findings with respect to the main published series on irradiated implant reconstructions.

### 3.4. Secondary Outcomes

#### 3.4.1. Salvage Autologous Reconstruction

No patient required conversion to pedicled or free flap reconstruction during the follow-up period. This finding is clinically relevant, as salvage autologous reconstruction is often necessary in irradiated implant-based reconstructions complicated by severe capsular contracture or implant loss. The absence of conversion in our cohort highlights the potential of fat grafting to support long-term implant maintenance.

#### 3.4.2. Oncologic Safety

No local recurrences were reported throughout the observation period, with a mean follow-up exceeding 7 years. While systemic relapses were beyond the scope of this study and were not analyzed, the lack of local recurrence is particularly reassuring given previous concerns regarding the potential tumor-promoting role of adipose-derived stromal cells.

#### 3.4.3. Patient Satisfaction

All patients were able to maintain their implant reconstruction at the last follow-up. Subjective reports indicated improvements in breast softness, pliability, and contour following fat grafting, which contributed to an overall perception of enhanced aesthetic outcomes. Although satisfaction was not measured with validated questionnaires, the consistency of these reports across the cohort supports the perceived clinical benefit of lipofilling in this setting.

## 4. Discussion

### 4.1. Impact of Radiotherapy on Implant-Based Reconstruction

Radiotherapy remains a major challenge in implant-based breast reconstruction. Ionizing radiation induces fibroblast proliferation, chronic inflammation, and disorganized collagen deposition, ultimately leading to capsular contracture and higher rates of reconstructive failure. Reported incidences range from 28% to over 50% in irradiated implants [7]. These figures are significantly higher than those observed in non-irradiated reconstructions, underscoring the detrimental effect of postmastectomy radiotherapy (PMRT) on implant outcomes.

Consequently, autologous reconstruction is often regarded as the gold standard for patients requiring PMRT, since vascularized tissue transfer provides a more resistant and biologically favorable environment [8,9,28]. Nonetheless, this approach is not universally feasible. Comorbidities, advanced age, limited donor-site availability, or patient refusal may preclude flap-based procedures. Furthermore, factors such as mastectomy flap thickness and vascularity have been shown to critically influence reconstructive outcomes, with thin or ischemic flaps predisposing to complications including necrosis, delayed healing, and eventual implant loss [9]. Recent reports have also suggested that the timing of reconstructive stages may influence long-term outcomes, as delayed implant exchange has been associated with a higher incidence of capsular contracture in irradiated reconstructions [29]

In parallel, the introduction of novel prosthetic adjuncts has expanded the reconstructive armamentarium, while prepectoral reconstructions using dermal slings or acellular dermal matrices (ADMs) are gaining popularity for selected patients [10,12]. These strategies aim to mitigate implant-related complications in irradiated fields, although long-term evidence remains limited.

Despite these advances, a significant subset of patients continues to experience unsatisfactory outcomes after PMRT. In this context, autologous fat grafting has emerged as a regenerative strategy with the potential to enhance tissue quality, improve aesthetic results, and reduce the incidence of capsular contracture [30,31].

### 4.2. Role of Fat Grafting as a Regenerative Strategy

Fat grafting has emerged as a valuable tool to counteract radiation-induced damage. Adipose-derived stromal cells (ADSCs) contained within the graft are capable of promoting angiogenesis, modulating fibrosis, and exerting immunomodulatory effects [32]. These biological mechanisms are particularly relevant in irradiated fields, where chronic inflammation, vascular damage, and fibrosis compromise tissue pliability and increase the risk of implant-related complications.

Clinical studies have consistently demonstrated that lipofilling can improve skin texture, elasticity, and overall tissue quality in irradiated breasts, with patients often reporting enhanced softness and pliability after treatment. Importantly, the regenerative potential of ADSCs has been linked not only to direct cellular effects but also to paracrine signaling that stimulates resident tissue repair and neovascularization [13,33].

Nevertheless, evidence regarding the role of fat grafting in the prevention of capsular contracture remains limited and sometimes conflicting. While several groups have reported a protective effect, reducing both the incidence and severity of contracture, others, such as Martin et al., failed to confirm this association in their retrospective series [14]. Variability in patient selection, timing of fat grafting relative to radiotherapy, and technical differences in harvesting and injection methods may explain the heterogeneity of outcomes across published studies.

### 4.3. Comparison with Literature

The present study demonstrated an incidence of capsular contracture of 18.8% at a mean follow-up of 7.7 years, which is markedly lower than the rates of 28–50% consistently reported in the literature for irradiated implants without fat grafting (Table 2). Although the study lacked an internal control group without fat grafting, the comparison with historical rates provides contextual insight. Published series of irradiated implant reconstructions without lipofilling report capsular contracture rates consistently above 35–50%, suggesting that the reduced incidence observed in our cohort may be at least partially attributable to the regenerative effect of fat grafting. However, this remains a hypothesis-generating observation that requires confirmation in comparative prospective studies. Notably, none of our patients developed severe Baker grade IV contracture, and no patient required salvage autologous reconstruction. These findings suggest that fat grafting may play a protective role in maintaining implant viability over the long term.

Our results are in contrast with those of Martin et al. [14], who did not observe a significant protective effect of lipofilling in irradiated implants. Several factors may explain this discrepancy. First, differences in patient selection are relevant: in our series, fat transfer was performed only in patients who had not developed acute radiation-induced complications and who demonstrated stable implant maintenance for at least one year after radiotherapy. This strict selection likely enriched our cohort with patients more likely to benefit from regenerative support while excluding those at higher risk of early failure.

It must be emphasized that our study did not include a non-fat-grafted control group. Therefore, the observed outcomes cannot be directly attributed to fat grafting alone. Although historical comparisons provide a contextual benchmark, they are inherently limited by differences in surgical technique, patient selection, and follow-up duration across studies. Consequently, the present findings should be interpreted as hypothesis-generating rather than conclusive evidence of a protective effect.

Second, variability in surgical technique and timing may also account for heterogeneous results across studies. Fat harvesting, processing, and injection methods are not yet standardized, and differences in cumulative grafted volume and number of sessions may significantly influence outcomes [34]. In our cohort, repeated small-volume grafting appeared sufficient to enhance tissue pliability and support long-term implant tolerance.

Finally, the relatively long follow-up in our series strengthens the reliability of the findings, as capsular contracture in irradiated patients often develops several years after treatment. Shorter follow-up periods, as in other reports, may underestimate the true incidence of contracture and its relationship with fat grafting.

### 4.4. Oncologic Safety

Concerns have been raised regarding the oncologic safety of lipofilling in breast cancer patients, particularly given the potential proangiogenic and immunomodulatory properties of ADSCs. Experimental studies have suggested that stromal vascular fraction cells may influence the tumor microenvironment, raising theoretical risks of promoting recurrence in previously treated breast tissue [35,36,37,38].

In our cohort, however, no cases of local recurrence were observed over a long-term follow-up period, supporting the safety of this approach in carefully selected patients. These findings are consistent with the growing body of clinical evidence, including large retrospective series and meta-analyses, which have demonstrated no significant increase in local or systemic recurrence rates after fat grafting in breast reconstruction. Importantly, oncologic surveillance in our series was conducted with regular clinical and radiological follow-up, further strengthening the reliability of these results.

Taken together, our data contribute to the reassuring evidence that lipofilling can be safely integrated into reconstructive pathways for breast cancer survivors, provided that strict patient selection criteria and adherence to oncologic follow-up protocols are respected.

### 4.5. Clinical Implications

Our data suggest that fat grafting may represent a valuable adjunct in maintaining implant-based reconstruction after radiotherapy. For patients who are not candidates for autologous reconstruction, lipofilling offers the potential to lower the risk of capsular contracture, improve the quality and pliability of irradiated soft tissues, and reduce the likelihood of requiring salvage procedures.

The clinical impact of these findings is twofold. First, by enhancing the biological environment around implants, fat grafting may help preserve reconstructive results in a subgroup of patients who would otherwise be at high risk of implant loss. This contributes directly to improved patient quality of life, as reconstruction failure is consistently associated with psychological distress, body image dissatisfaction, and the need for more invasive corrective surgery.

Second, reducing the incidence of late complications and revision procedures carries important health-economic implications. Implant failure after radiotherapy often necessitates complex salvage operations, typically involving autologous flaps, which are associated with higher surgical morbidity, longer recovery, and increased costs for the healthcare system. By potentially minimizing the need for these interventions, lipofilling may represent not only a biologically advantageous but also a cost-effective strategy within the reconstructive pathway.

### 4.6. Limitations and Future Directions

This study has several limitations. Its retrospective design inherently carries risks of selection and information bias, while the relatively small sample size limits the statistical power and generalizability of the results. The strict inclusion criteria, although ensuring a homogeneous cohort and long-term follow-up, inevitably reduce external validity and may have selected for patients with more favorable baseline conditions.

Another limitation lies in the assessment of patient satisfaction, which was performed through clinical interviews rather than validated questionnaires such as the BREAST-Q. As a result, patient-reported outcomes could not be quantified or compared with other series using standardized tools. Moreover, the cohort included mostly normal-weight patients with relatively small implant volumes, which may have contributed to the low incidence of complications. These factors limit the generalizability of the findings to populations with higher BMI or larger prostheses. Furthermore, our findings cannot be extrapolated to patients experiencing severe early radiation-induced complications, who were excluded from this analysis and may represent a higher-risk subgroup.

Future prospective studies with larger and more diverse patient cohorts are needed to confirm these preliminary observations. Standardization of fat grafting protocols—including harvesting, processing, and injection techniques—would also help reduce heterogeneity across studies and clarify the true impact of lipofilling on capsular contracture prevention. Finally, radiological and histological analyses of the periprosthetic capsule could provide valuable insight into the biological mechanisms underlying the observed benefits of fat grafting, bridging clinical outcomes with basic science evidence.

## 5. Conclusions

Fat grafting appears to be a safe and effective adjunct in implant-based breast reconstruction after radiotherapy. In our long-term series, lipofilling was associated with a lower incidence of capsular contracture, no cases of severe contracture, and no need for salvage autologous reconstruction. Importantly, no local recurrences were observed, supporting the oncologic safety of this approach.

These findings highlight the potential of lipofilling not only as a regenerative tool but also as a strategy to reduce the physical, psychological, and economic burden of reconstructive failure.

However, the absence of a control group, the small sample size, and the predominantly low-BMI population require that these results be interpreted with caution. For carefully selected patients who are not candidates for autologous reconstruction, fat grafting may help preserve implant viability and improve the quality of irradiated tissues, but further prospective studies with standardized protocols are needed to confirm these preliminary observations.

## Figures and Tables

**Table 1:** Clinical, intraoperative data.

Category	Data
**Number of patients**	32
**Follow-up (years):** Mean	7.7
**BMI (kg/m^2^):** Mean, Standard Deviation	23.8 ± 2.3
**Age (years):** Mean, Standard Deviation	56.1 ± 10.8
**Implant volume (cc):** Mean, Standard Deviation	335.9 ± 38.5

**Table 2:** Reported incidence of capsular contracture in irradiated implant-based breast reconstructions: comparison with the present study.

Author/Year	Study Design	N (Patients)	Follow-Up (Months)	Capsular Contracture Rate	Notes
Nava et al., 2011 [7]	Prospective cohort	159	50	53.3% (irradiated TE group)	TE irradiated group; part of larger study
Cordeiro et al., 2014 [6]	Prospective cohort (single surgeon)	1415 (319 irradiated)	54.4	6.9% grade IV (irradiated group)	Only PMRT to permanent implant
Reish et al., 2015 [8]	Retrospective cohort	296	60	39%	Includes ADM and RT; multivariate analysis
Ricci et al., 2017 [5]	Systematic review & meta-analysis	2348	39.5	37.5%	Pooled data from 5 studies (RT + implant)
Martin et al., 2021 [14]	Retrospective comparative cohort	57	23.5	42.1% overall, 58.3% Baker III/IV	AFG vs. no AFG; no protective effect found
**Present study (2025)**	**Retrospective**	**32**	**92.4**	**18.8%**	**All patients received lipofilling after RT**

## Data Availability

The data presented in this study are available from the corresponding author on reasonable request. Restrictions apply to protect patient privacy and comply with institutional and national regulations.
